# Supplementary material for: Real-time observation of the isothermal crystallization kinetics in a deeply supercooled liquid
Source: Sci Rep. 2017 Mar 3;7:43671. doi: 10.1038/srep43671 (PMC5334641; doi:10.1038/srep43671)
Supplement: Supplementary Information [file srep43671-s1.pdf]

Supplementary information: Real-time observation of the isothermal crystallization kinetics in a deeply supercooled liquid

M. Zanatta,[1,2,*] L. Cormier,[3] L. Hennet,[4,5] C. Petrillo,[6,7] and F. Sacchetti[6,7]

[1]*Dipartimento di Informatica, Università di Verona, I-37134 Verona, Italy*
[2]*ISC-CNR c/o Dipartimento di Fisica, Sapienza Università di Roma, I-00185 Roma, Italy*
[3]*Institut de Minéralogie, de Physique des Matériaux, et de Cosmochimie (IMPMC), Sorbonne Universités, UPMC Université Paris 06, CNRS UMR 7590, Muséum National d'Histoire Naturelle, IRD UMR 206, F-75005 Paris, France*
[4]*Conditions Extrêmes et Matériaux: Haute Température et Irradiation, CEMHTI-CNRS, Université d'Orléans, F-45071 Orléans, France*
[5]*Laboratoire Léon Brillouin, CEA-CNRS, CEA Saclay, F-91191 Gif sur Yvette, France*
[6]*Dipartimento di Fisica e Geologia, Università di Perugia, I-06123 Perugia, Italy*
[7]*IOM-CNR c/o Dipartimento di Fisica e Geologia, Università di Perugia, I-06123 Perugia, Italy*

Instrument resolution

The instrument resolution function Δ was estimated by considering the width of the Bragg peaks in KBr powder. This material has a fcc structure with a lattice parameter $a = 6.598$ Å that provides peaks even at relatively low scattering angles. In particular, for $\lambda = 0.724$ Å, the (111) reflection is at $2\theta = 10.9°$, a value that ensures a reliable determination of the instrument resolution also in the region of the first peaks of the α-quartz GeO_2.

The sample was loaded into an Al cylindrical cell and measured at $T = 373$ K to avoid any water contamination of the KBr powder. Data were analyzed according to Refs. [S2, S3]. The empty cell contribution was not subtracted and Al Bragg peaks were thus included in the analysis. The so-obtained diffraction pattern is shown in Fig. S1.

The measured intensity was fitted as the sum of three components

$$S(2\theta) = S_T(2\theta) + S_B(2\theta) + bkg. \tag{S1}$$

The first term $S_T(2\theta)$ accounts for the thermal diffuse scattering, $S_B(2\theta)$ represents the Bragg peaks pattern of KBr and Al, whereas bkg is a flat background due to the incoherent contributions. The TDS contribution $S_T(2\theta)$ was estimated using a very simple approximation from Ref. [S1], i.e.

$$S_T(2\theta) = 1 - e^{-2W}, \tag{S2}$$

where $\exp(-2W) = \exp\left(-2B(\frac{\sin\theta}{\lambda})^2\right)$ is the Debye Waller factor. The value $B = (2.33 \pm 0.09)$ Å^2 is taken from Ref. [S4]. The Bragg peaks contribution is written as a sum of Gaussian functions and it turns out to be:

$$S_B(2\theta) = \sum_i \frac{A_i}{\sigma_i\sqrt{2\pi}} e^{-\frac{1}{2}\left(\frac{2\theta - 2\theta_i}{\sigma_i}\right)^2}. \tag{S3}$$

---

*Electronic address: marco.zanatta@univr.it

The position of the ith peak $2\theta_i$ is given by the structure, whereas its area A_i is fitted independently for each peak. Conversely, we assume that the full width at half maximum of each peak is completely given by the instrument resolution Δ. Following Ref. [S5] we can write that

$$\Delta = \sqrt{W_2 \tan(\theta)^2 + W_1 \tan(\theta) + W_0}, \tag{S4}$$

where W_2, W_1 and W_0 depend on the instrument collimation and they can be obtained by fitting Eq. S1 to the data.

Fig. S1 shows the results of the fit on KBr data. The fit was refined by including both KBr and Al peaks, red and black lines in Fig. S1(b) respectively. The instrument resolution determined according Eq. S4 is reported in the inset of Fig. S1.

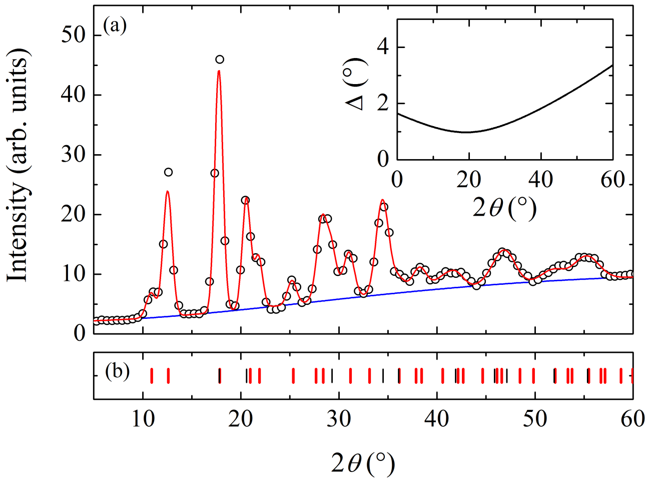

FIG. S1: (a) Diffraction pattern measured on KBr powder. Experimental data (open black circles) are fitted by using the model of Eq. S1 (red line); the blue line is the sum of $S_T(2\theta)$ and bkg. The instrumental resolution Δ as obtained by the fit is reported in the inset (black line). (b) Positions of the Bragg peaks used in the fit; thick red stiks mark the KBr structure whereas the blue ones represent the position of Al peaks due to the container.

Johnson-Mehl-Avrami-Kolmogorov analysis

The Johnson-Mehl-Avrami-Kolmogorov (JMAK) model [S6–S10] describes the time evolution of the fraction of transformed material during an isothermal crystallization. For an isothermal transformation, the JMAK equation can be written as

$$X(t) = 1 - e^{-kt^n}, \tag{S5}$$

where $X(t)$ is the fraction of transformed volume and k an effective rate constant depending on the nucleation and growth rates. The exponent n is termed Avrami exponent and is expected to assume integer or half an integer values. The Avrami exponent depends on the characteristics of the process. As an example, for continuous nucleation and 3D spherical growth, the Avrami exponent is $n = 4$. The JMAK equation implies a complete crystallization of the amorphous medium, which appears in contrast with the observed time evolution of the crystallized fraction A_c. Figure S2 shows a fit of A_c with Eq. S5 using k and n as free

parameters. The results is represented with a blue dashed line and it does reproduce neither the shape nor the long-time behavior.

In order to improve the agreement at long t, we can introduce a constant scale parameter X_c accounting for the incomplete crystallization. Consequently, Eq. S5 can be rewritten as

$$X(t) = X_c \left[1 - e^{-kt^n} \right] . \tag{S6}$$

The result of the fit with this modified JMAK equation is shown in Fig. S2 (solid blue line). The scale parameter results $X_c = 0.76 \pm 0.01$ while the Avrami exponent is $n = 1.92 \pm 0.02$. Equation S6 provides a considerably better agreement than the JMAK equation, in particular in the first stages of the crystallization processes. However, as the crystallized fraction increases, the model is still not able to accurately reproduce the shape of A_c as the approach proposed in the paper.

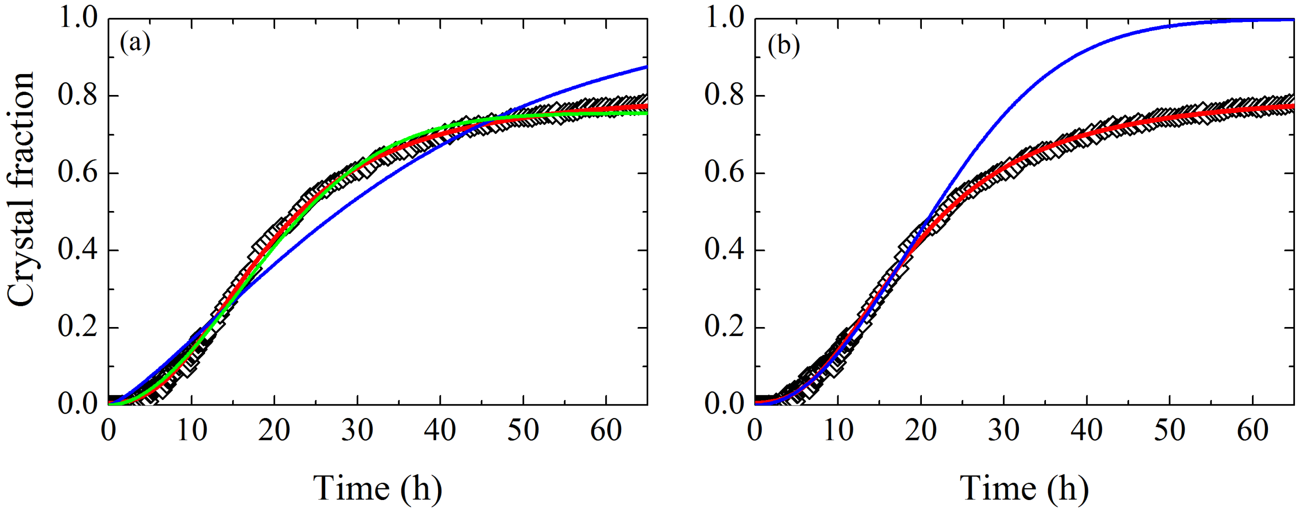

FIG. S2: (a) Time evolution of the crystalline fraction A_c (black diamonds). The solid red line represents the fit with the model presented in the text. The solid blue line is the fit with the JMAK equation, Eq. S5, whereas the solid green line is obtained by using Eq. S6. (b) Time evolution of the crystalline fraction A_c (black diamonds) fitted with the Eq. S5 considering only the first about 20 h of the process (solid blue line). For comparison, the fit obtained with the model described in the text is also reported (solid red line).

Figure S2(b) shows a fit with the JMAK model obtained by considering only the first about 20 h of the process. Within this range, the JMAK equation provides a very good description of the experimental data. As a matter of fact, at the beginning of the crystallization process, the hypotheses of random nucleation and similar growth of spherical regions hold. Increasing the transformed fraction in a non-diffusive environment ($T \ll T_m$), the density difference between crystallized and amorphous region influences both the nucleation and the growth mechanism, thus producing the observed non-complete crystallization.

[S1] Warren, B.E. *X-Ray Diffraction*, 2nd Ed. (Dover Publications, Inc., New York, 1990).

[S2] Petrillo, C., & Sacchetti, F. Analysis of neutron diffraction data in the case of high-scattering cells. Acta Crystallogr., Sect. A: Found. Crystallogr. **46**, 440 (1990).

[S3] Petrillo, C., & Sacchetti, F. Analysis of neutron diffraction data in the case of high-scattering cells. II. Complex cylindrical cells. Acta Crystallogr., Sect. A: Found. Crystallogr. **48**, 508 (1992).

[S4] Butt, N.M., Ahmed, N., Beg, M.M., Atta, M.A., Aslam, J., & Khan, Q.H. The Debye-Waller factor of KBr by powder elastic neutron diffraction. Acta Crystallogr., Sect. A: Found. Crystallogr. **32**, 674 (1976).

[S5] Caglioti, G., Paoletti, A., & Ricci, F.P. Choice of collimators for a crystal spectrometer for neutron diffraction. Nucl. Instrum. Methods **3**, 223-228 (1958).

[S6] A.N. Kolmogorov, On the statistical theory of the crystallization of metals, Isz. Akad. Nauk SSR, Ser. Fiz. **3**, 355 (1937).

[S7] W.A. Johnson and R. Mehl, Reaction kinetics in processes of nucleation and growth, Trans. AIME **135**, 416 (1939).

[S8] M. Avrami, Kinetics of Phase Change. I General Theory , J. Chem. Phys. **7**, 1103 (1939).

[S9] M. Avrami, Kinetics of Phase Change. II Transformation?Time Relations for Random Distribution of Nuclei, J. Chem. Phys. **8**, 212 (1940).

[S10] M. Avrami, Granulation, Phase Change, and Microstructure Kinetics of Phase Change. III, J. Chem. Phys. **9**, 177 (1941).
